# Methods for safely sharing dual-use genetic data

**DOI:** 10.3389/fmicb.2026.1716431

**Published:** 2026-02-11

**Authors:** Sterling Sawaya, Chien-Chi Lo, Po-E Li, Blake Hovde, Patrick Chain

**Affiliations:** 1 GeneInfoSec Inc., Boulder, CO, United States; 2 Los Alamos National Laboratory, Los Alamos, NM, United States

**Keywords:** cyberbiosecurity, DNA sequencing security, genome sequencing, information security, pathogen sequencing

## Abstract

**Background:**

Some genetic data has dual-use potential. Sharing pathogen data has shown tremendous value. For example therapeutic development and lineage tracking during the COVID pandemic. This data sharing is complicated by the fact that these data have the potential to be used for harm. The genome sequence of a pathogen can be used to enable malicious genetic engineering approaches or to recreate the pathogen from synthetic DNA. Standard data security methods can be applied to genetic data, but when data is shared between institutions, ensuring appropriate security can be difficult. Sensitive data that is shared internationally among a wide array of institutions can be especially difficult to control. Methods for securely storing and sharing genetic data with potential for dual-use are needed to mitigate this potential harm.

**Results:**

Here we propose new methods that allow genetic data to be shared in a data format that prevents a nefarious actor from accessing sensitive aspects of the data. Our methods obfuscate raw sequence data by pooling reads from different samples. This approach can ensure that data is secure while stored and during electronic transfer. We demonstrate that by pooling raw sequence data from multiple samples of the same organism, the ability to fully reconstruct any individual sample is prevented. In the pooled data, most genomic information remains, but reads or mutations cannot be directly attributed to any individual sample. To further restrict access to information, regions of a genome can be removed from the reads.

**Conclusion:**

Our methods obscure genomic information within raw sequence reads. This method can allow genetic data to be stored and shared while preventing a nefarious actor from being able to perfectly reconstruct an organism. Broad-scale sequence information remains, while fine scale details about specific samples are difficult or impossible to reconstruct. Our software is available at https://github.com/Geneinfosec-Inc/ReadMixer.

## Background

As biotechnology advances, there is an increased potential that it can be misused (Smith and Sandbrink, 2022). Today, methods in synthetic biology can facilitate the creation of novel organisms. DNA can be accurately synthesized and this DNA can be inserted into an organism using CRISPR/CAS9 (Doudna and Charpentier, 2014). Entire portions of a genome can either be directly synthesized or be rewritten to become any desired sequence. Furthermore, entire organisms can be constructed with synthetic DNA (Gibson et al., 2010). The ability to fully reconstruct a virus or bacteria with only a genome sequence has been demonstrated (Mitchell and Ellis, 2017). Although today this method is more cumbersome and expensive than simply adding or deleting genes from a genome, as synthetic biology continues to advance, these capabilities will become easier and more widespread.

The ability to recreate an organism with genetic data introduces a dilemma for those generating pathogen genome data. There is a need to share genetic information of pathogens so that their origin and evolution can be understood, and also a beneficial use of the genetic information when designing countermeasures to an infectious disease (Baker et al., 2020; Neves et al., 2023). However, because this data can be misused, there may be hesitance to share genetic data that has been collected (Dos S Ribeiro et al., 2018), or perhaps even withhold publishing to avoid pressure to share the data. For genetic data from the most dangerous pathogens, sharing data may be prohibited in some countries, especially if the data is shared internationally (Edelstein and Sane, 2015; Edelstein et al., 2018).

There are international treaties for sharing specific pathogen data, such as the Pandemic Influenza Preparedness Framework (Thompson et al., 2006). This framework encourages the sharing of genomic data from influenza, but is, however, not legally binding and its enforceability has never been tested (Rourke and Eccleston-Turner, 2021). There are ongoing negotiations to expand this approach to other pathogen data, but in general the sharing of pathogen sequence data only occurs between countries through bilateral agreements (Rourke, 2019). The ownership of genetic data and the sharing of benefits or products from genetic data are governed by the Nagoya protocol, but this treaty requires a case-by-case bilateral negotiation to make specific arrangements (Buck and Hamilton, 2011).

The standards and regulations for data sharing during a world-wide crisis have been tested by the COVID pandemic. During the initial stages of the outbreak, sharing of pathogen sequence data was essential for tracking its spread, predicting how the virus may evolve, and developing countermeasures such as mRNA vaccines (Chiara et al., 2020; Rockett et al., 2020). Without the prompt sharing of accurate sequence data, the world may not have been able to respond rapidly to this novel disease. Nevertheless, as data was openly shared, concerns about equity and benefit-sharing arose (Pratt and Bull, 2021; Shu and McCauley, 2017). These and other concerns can restrict fully open pathogen genomic data sharing during disease outbreaks.

The open sharing of scientific data stands as an ideal standard on which major scientific advances rely (Huston et al., 2019; Murray-Rust, 2008). However, this data sharing approach can conflict with the data sharing restrictions in place for the genomic data of certain pathogens. Currently, the standard method by which critical data from dangerous pathogens can be shared involves data sharing platforms with various levels of access control. While frameworks like GISAID (Shu and McCauley, 2017) and the PHA4GE Data-Sharing Accord (Griffiths et al., 2022) facilitate managed sharing by protecting submitter rights, and INSDC prioritizes “free and unrestricted access” (Karsch-Mizrachi et al., 2025), none of these platforms technically prevent the reconstruction of dangerous pathogens by actors with legitimate access. Consequently, pathogen data is either siloed, so no genetic data is shared, or the data is wholly shared, allowing anyone with access to the data to recreate the pathogen (Vinatzer et al., 2019). New, safer methods for accessing the sequence data of pathogens are needed. The methods proposed here provide a bridge between fully replicable data and data that remains entirely hidden in silos, enabling data sharing for legitimate research purposes while preventing pathogen reconstruction.

The methods here are inspired by techniques in molecular cryptography, in which cryptographic protocols are applied directly to DNA molecules to ensure that sensitive information is protected before the DNA is sequenced. Molecular cryptography can be used to mix DNA samples in such a way that a key is needed to determine which read from a DNA sequencer belongs to which sample (Sawaya, 2017). Without the key, data from the resulting pool is obfuscated. A similar approach can be done with software, in which reads from multiple files are pooled together and the meta-data is stripped. This approach utilizes the pooling breakthrough that allows high-throughput methods to scale next-generation sequencing but here we intentionally do not allow the data in the pool to be directly attributed to an individual sample. Using software, we examine the use of this approach. To produce output that is similar to molecular cryptography, our software adds a random string of nucleotides (the keys) to the sequence identifier line in the FASTQ file. The software also strips the sequence identifier line of any previous information and produces a key file that contains the keys tied to their original sequence identifier line and FASTQ file name. Here, we propose that this new method allows for sharing genetic sequence data while protecting sensitive aspects of the data.

## Materials and methods

**FIGURE 1:**
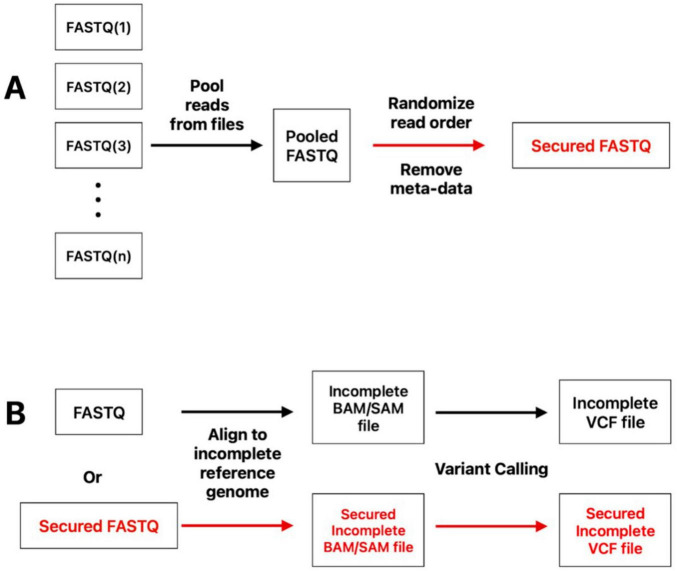
Workflow for methods used to obfuscate genomic data. **(A)** Method for pooling reads to generate a secure FASTQ file. FASTQ files were pooled and their order randomized. **(B)** Method for removing genomic region(s) to limit information within a set of (pooled) samples. Samples, or a pool of samples from panel **(A)**, are aligned to an incomplete reference genome to produce a file that lacks complete genomic information.

We developed new methods for formatting genetic data that prevents anyone with access to the data from being able to reconstruct an entire genome (Figure 1). The first proposed method involves the following (Figure 1B): DNA sequencing reads from a sample (or group of samples) are aligned to an incomplete reference genome. A substantial portion of this genome must be removed (e.g., removal of a full gene). The resulting alignment file (e.g., in SAM format) can then be processed into a variant call file (VCF) or can be shared directly. The aligned file or the VCF will be insufficient to recreate the full genome of the sample, as sensitive sequence regions that were removed are not included in the alignment of assembly files.

Our second proposed method is more sophisticated, and can occur prior to the application of the first proposed method (Figure 1A). Here, we pool DNA sequence reads from multiple FASTQ datasets that originate from DNA samples of the same organism and the metadata from each read is stripped. The order of the reads in the pooled data is then randomized such that reads that originate from an individual sample are distributed throughout the pool. The resulting file remains in FASTQ format and contains all of the reads from the data of the original samples but paired-end read information is lost. The files with pooled reads can be directly processed into VCF files by mapping reads to a reference genome and calling variants. For additional security, the pooled data can also be processed with the first proposed method (aligning to an incomplete reference genome). This process also strips sample information from individual reads, adds a random identifier, and creates a separate mapping file (key file) that can be used to reverse the process. The software implementation of this pooling method is publicly available at https://github.com/Geneinfosec-Inc/ReadMixer.

To examine the read pooling method, we applied it to FASTQ data from SARS-CoV-2, from Monkeypox virus (MPXV), and from *Bacillus anthracis*. For SARS-CoV-2, we generated 7 independent datasets in FASTQ format, consisting of a unique mixture of 2, 5, 10, 50, 100, and 500 sequencing datasets obtained from CDC (see Supplementary Table 1). For *B. anthracis* we generated 4 distinct datasets, consisting of 2, 5, 10 and 50 isolates, randomly subselected from National Center for Biotechnology Information (NCBI) Sequence Read Archive (SRA). (see Supplementary Table 2) with search criteria “Organism = *Bacillus anthracis*” and “platform = Illumina” and “layout = paired.” Four distinct Monkeypox virus (MPXV) datasets were generated by pooling 2, 5, 10, and 50 isolates, each randomly selected from the 2,119 NCBI SRA samples (see Supplementary Table 3).

The metadata from each dataset, including the depth of coverage and strain variants/lineages, were utilized as the ground truth for the composition/abundance of each pooled dataset. For SARS-CoV-2, the EDGE COVID-19, build 20230131 (Lo et al., 2022), was used for variant calling and lineage assignment was done with Pangolin (v4.3.1) (O’Toole et al., 2021). For *B. anthracis*, the EDGE, build 20231115 (Li et al., 2017) was used for read-mapping to the reference genome *Bacillus anthracis* str. “Ames Ancestor” (AE017334.2) and for variant calling.

Freyja v1.4.8 (Karthikeyan et al., 2022) was used for deconvoluting the variants/lineages in the datasets from the VCF files from the pooled SARS-CoV-2 data. This program was designed to deconvolute genomes for “mixed” samples, with the ability to predict the SARS-CoV-2 lineages present within the mixed samples. We used Freyja to predict the proportion of SARS-CoV-2 variants and lineages in the datasets, for both pooled data and for individual datasets. We also bootstrapped the reads from the pool of 2 and 10 datasets, with 1000 replicates, and ran Freyja on these bootstrapped pools to predict strain variants.

To examine the pooled *B. anthracis* and MPXV data, we generated variant frequency heatmaps using the Plotly dash-bio.Clustergram package (Plotly, 2024). The Euclidean pairwise distance (Dokmanic et al., 2015) and complete linkage clustering algorithms (Großwendt and Röglin, 2017) were employed to the variant calling result based on variant locations. By applying these algorithms to the variant calling results, we can identify groups of variants with similar frequency patterns across different genomic locations. This clustering approach helps to identify, visualize, and interpret the relationships between variants in the *B. anthracis* and MPXV data, potentially revealing insights into genetic similarities or differences among the different treatments.

## Results

By examining pooled data for larger and larger pools of sequencing datasets (FASTQ reads), we tested the ability of our pooling method to obfuscate genomic data using established bioinformatics methods. We estimated the composition of the pooled data using these methods and compared the results to the true composition based on the samples used to generate those pools.

**FIGURE 2:**
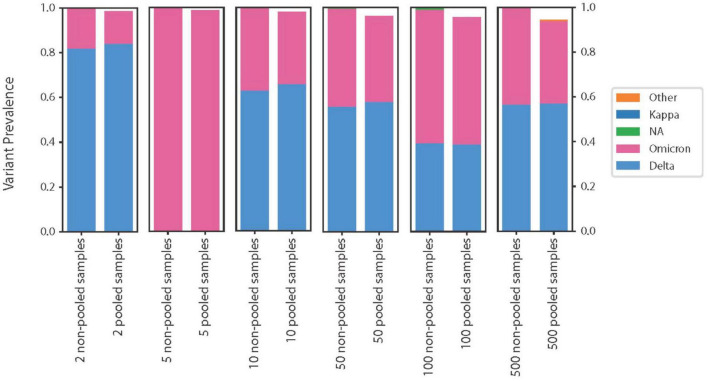
Prediction of variants present for each sample and for the pooled datasets. For each group examined, Freyja was used to predict variants present within each individual sample (left stacked histogram in each subplot), and the proportion of each variant in the pooled sample represents the ratio of reads from that sample used in the pooled dataset. The right stacked histogram for each subplot represents the variants present within the pooled datasets as predicted by Freyja.

For the SARS-CoV-2 samples, the EDGE COVID-19 workflow was used to provide majority-rule based variant calls (Lo et al., 2022). Consequently, regardless of whether the SARS-CoV-2 reads were pooled or whether datasets were analyzed separately, the same single nucleotide polymorphism variants (SNVs) and insertion/deletion (INDELs) were found. Freyja, another tool designed for SARS-CoV-2 (Karthikeyan et al., 2022), provides a population level analysis of the pooled reads. Freyja was successfully able to estimate the approximate proportion of variants present within the pooled data (Figure 2). Note that the samples used to generate the pool of 5 datasets did not contain any Delta variant, being entirely composed of the Omicron variant, so a comparison of variant calling was not possible with this group.

Freyja demonstrated a slight bias when predicting the fraction of the variant in the pool, with a tendency to estimate a slightly higher fraction of the common variant. We bootstrapped the pool of 2 and 10 datasets for 1000 replicates to examine the variance in this trend. We found this trend also existed in the bootstrapped pools. The mean fraction of Delta in the pools of 2 and 10 were 0.84 and 0.66, while the bootstrapped values were 0.856 ± 0.007 and 0.68 ± 0.01, respectively.

**FIGURE 3:**
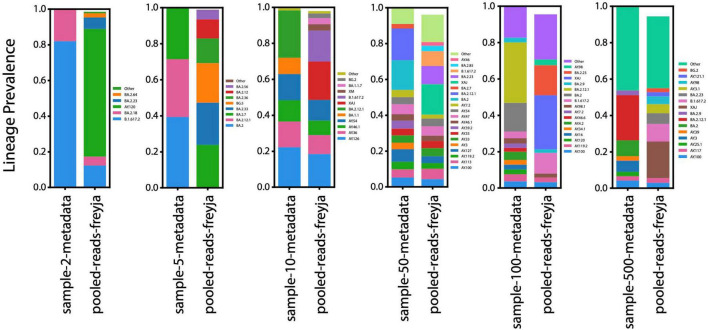
Prediction of lineages present within samples and within the pooled datasets. For each group examined, Freyja was used to predict lineages present within each individual dataset (left stacked histogram in each subplot), and the proportion of each lineage in the pooled dataset represents the ratio of reads from that dataset used in the pooled dataset. The right stacked histogram for each subplot represents the lineages present within the pooled datasets as predicted by Freyja. Only higher frequency variants are shown and as the pool grows larger there are too many variants to accurately plot. These are plotted as “other” and represent a larger portion of the pool as the number of datasets grows.

While variants were accurately detected by Freyja, the lineages identified were not always correct (Figure 3). The prediction of lineages in Freyja produced primarily false positives, even when only two datasets were pooled. Freyja’s ability to predict the presence of lineages in pooled data had diminishing accuracy as more datasets were pooled together. Notably, Freyja tended to overestimate the number of lineages in the pooled data, and this overestimate increased as more datasets were pooled together.

While the community is familiar with delineating variants in viral outbreaks, the definition of a lineage within bacterial species is not as straightforward. Due to the lack of sublineage definitions, and also, since there exists no similar program to Freyja for bacteria, we took a different approach to demonstrate that our method provides obfuscation of bacterial genomes.

**FIGURE 4:**
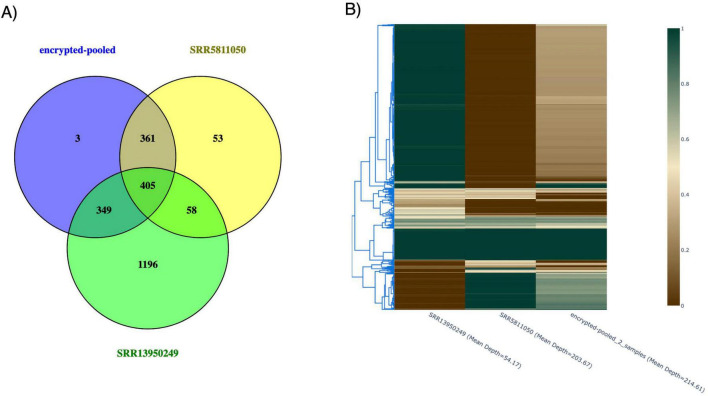
Pooled *B. anthracis* 2-samples SNVs and individual sample SNVs. **(A)** Venn diagram (https://bioinfogp.cnb.csic.es/tools/venny/). The variants present within two individual accessions (SRR5811050 and SRR13950249) were compared to determine which variants overlap between the different accessions and also between the variants called in the pooled accessions. **(B)** Heatmap. *Y*-axis represents SNV position clustering. Heatmap color represents the SNVs ratio which is defined as the alternate base versus the reference base ratio.

For *B. anthracis* we generated 4 distinct datasets, consisting of 2, 5, 10 and 50 isolates, for a total 67 SRA sequencing datasets (accessions for whole genome sequences). When the 67 individual sequencing datasets of *B. anthracis* were individually mapped to the reference genome, an average genome coverage ranging from 97.04% to 99.06% was achieved. The variant calling analysis revealed a SNV count ranging from 8 to 2,653. To assess the pooled *B. anthracis* data, we compared the SNV positions in the pools with those from individual sequencing datasets. Figure 4A presents a Venn diagram illustrating the SNVs found in the pooled 2-sequencing datasets relative to those found in the individual sequencing datasets alone (SRR5811050 with a mean depth 54× and SRR13950249 with a mean depth 203×). The pooled sequencing datasets share 1,115 SNVs with the individual sequencing datasets. However, the pooling of two sequencing datasets introduced three false positive SNVs that were not present in the individual sequencing datasets analyses. These false positives occurred because, although the alternative base ratios in the individual sequencing datasets were below the detection threshold at these positions, their combined ratios in the pooled datasets exceeded the threshold and were detected by the variant calling algorithm. Conversely, each individual dataset contains 1,307 SNVs that were not detected in the pooled data, representing a high false negative (FN) rate in the pooled analysis. This high rate is likely due to two factors: (1) pooling increased the depth coverage of non-variant regions, diluting the signal of true variants (1,196 FN in SRR13950249 and 53 FN in SRR5811050), and (2) the pooling method removed paired-end information, preventing the mapping algorithm from rescuing reads that did not perfectly align due to SNVs (58 FN shared by two individual datasets). Figure 4B depicts a cluster analysis heatmap of the pooling of 2 datasets, along with data from the two individual datasets. The vertical dendrogram on the left of Figure 4B illustrates the hierarchical clustering of the datasets, revealing patterns of similarity. The heatmap itself shows variations in alternate base ratios across different SNV positions, with color gradients indicating the frequency of these variants. As seen in Figure 4B, although the SNV positions in the pooled datasets overlap with those in the individual datasets, there are differences in the alternative base ratios for other SNV positions. Notably, the top cluster highlights a “dilution” effect for false negative SNVs, where pooling reduces variant signals below detection thresholds. This result demonstrates that combining reads from different datasets can obscure variant information, leading to both false positives and false negatives.

**FIGURE 5:**
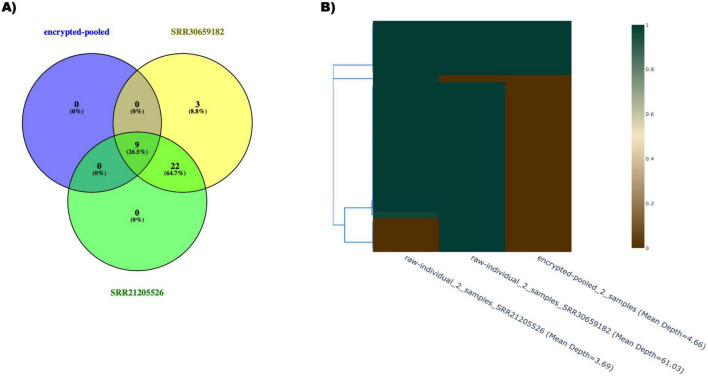
Pooled MPXV 2-samples SNVs and individual sample SNVs. **(A)** Venn diagram (https://bioinfogp.cnb.csic.es/tools/venny/). The variants present within two individual accessions (SRR30659182 and SRR21205526) were compared to determine which variants overlap between the different accessions and also between the variants called in the pooled accessions. **(B)** Heatmap. *Y*-axis represents SNV position clustering. Heatmap color represents the SNVs ratio which is defined as the alternate base versus the reference base ratio.

The Monkeypox virus analysis further demonstrates how pooling reads effectively obscures sample-specific genomic signals in organisms whose genome sizes fall between SARS-CoV-2 (∼30 kb) and bacteria (∼5 Mb). MPXV’s 197-kb-long double-stranded DNA genome provides an intermediate test case, enabling evaluation of how pooling behaves for large viral genomes that exhibit moderate genomic complexity relative to RNA viruses. As shown in the 2-sample Venn diagram (Figure 5A), all variants detected in the pooled sample were also present in the contributing isolates. Notably, the pooled dataset contained no SNVs unique to the pool, indicating that, unlike the *B. anthracis* case, the pooling procedure did not generate false-positive variants that exceed detection thresholds. This behavior is consistent with MPXV’s relatively low mutation rate and more homogeneous variant landscape (Scarpa et al., 2022), which reduce the likelihood of low-frequency alleles combining to form artifact variants.

Despite the absence of pool-specific SNVs, the heatmap and clustering profile (Figure 5B) show that variant-frequency patterns in the pooled MPXV sample are substantially altered relative to the individual isolates. The pooled profile does not cluster tightly with either isolate and shows clear dilution of alternate-base ratios, demonstrating that quantitative variant signals are still disrupted by the pooling process. As a result, although the pool does not introduce artificial mutations, it nonetheless obfuscates isolate-specific genomic information and impedes accurate reconstruction of individual MPXV genomes. These results confirm that pooling-based data security is effective across genome sizes from small RNA viruses to large DNA viruses and bacteria, even when variant counts are limited and false positives do not arise.

## Discussion

The data generated by our software has a similar format to a real-life scenario in which the read data from individual samples is not clear. Samples can contaminate each other, multiple variants of a disease can co-infect a patient, or a new, recombinant lineage can arise. The program Freyja is designed to disentangle these potential scenarios for SARS-CoV-2, making it an ideal approach by which we can test the limits of our method. Freyja was unable to accurately predict which SARS-CoV-2 lineages were present within our pooled data, indicating that our method successfully obfuscates lineage information (Figure 3). Consequently, our pooling method can prevent the reconstruction of complete SARS-CoV-2 genomes, even when only a few genomes are pooled. Freyja’s ability to accurately estimate the fraction of variants in our pooled data, on the other hand, indicates that our method does not conceal lineage-specific information (Figure 2). Recent work has shown that pooled whole-genome sequencing is an effective and inexpensive strategy for SARS-CoV-2 surveillance (Park et al., 2025). Our results demonstrate that this same pooling principle can be leveraged for biosecurity purposes, obfuscating individual genomes while preserving the population-level signal needed for surveillance.

When constructing genomes, many factors can influence variant calling. We expected that the differences between the bacterial and viral genomes examined here would influence how well our pooling method would obfuscate the genomes. Bacterial genomes are larger and more structurally complex than viral genomes and contain more variation, and thus the reconstruction of their genomes can be more difficult. Furthermore, delineation of bacteria lineages can be complicated by frequent horizontal gene transfer from distant species and large-scale genome rearrangements (Power et al., 2021; Smith and Andam, 2021). Therefore, we expected *a priori* that reconstructing *B. anthracis* genomes from the pooled sequence data would be a bigger challenge than for SARS-CoV-2. Without a custom tool like Freyja to disentangle genomes, our analysis instead focused on a comparison of variant calling between pooled data and individual datasets.

For data pooled from two *B. anthracis* datasets, the SNPs called in the pool did not always match the SNPs independently called from the two original datasets (Figures 4A, B). These results demonstrate that pooling data obfuscates variant calling. Accurate variant calling is needed to fully reconstruct genomes or to predict the presence of specific bacterial lineages. This effect becomes more pronounced as additional datasets are pooled (see Supplementary Figures 1–6), making it increasingly difficult to accurately call variants and fully reconstruct genomes. Note, however, that smaller regions of the genome, such as pathogenicity islands (Juhas et al., 2009), would be more readily reconstructed than whole genomes. This issue can be addressed by removing high-risk loci through an alignment to an incomplete reference genome (Figure 1B).

Similar to *B. anthracis*, the obfuscation from pooling MPXV datasets increases as the number of datasets increases (see Supplementary Figures 1–6). At 200 kilobases in size, the MPXV genome represents an intermediate between the small SARS-CoV-2 genome (∼50 kilobases) and the large, relatively stable genome of *B. antracis* (∼5 megabases). Our method’s ability to obfuscate the MPXV genomes demonstrates the effectiveness of our method on intermediate genome sizes and mutation rates, even when only 2 datasets were pooled. These results have practical applications. The MPXV recently caused a multi-country outbreak, prompting concerns about its pandemic potential. Consequently, methods that enable secure sharing of MPXV genomic data for surveillance while preventing misuse are of immediate public health relevance (Scarpa et al., 2022).

Overall, these results demonstrate that by pooling reads from the same species, the samples’ genomes become obfuscated. Not all information is concealed, however. Much of the genomic information remains: the species being sequenced, the common variants that are present in the pool, and general information about gene sequences. We also found that pooling more datasets always increases genome obfuscation, and larger genomes with more variants are more difficult to accurately reconstruct. However, the limits of this method have not been tested here. Pooling genomes of distantly related organisms will facilitate the attribution of a specific read to a specific lineage, limiting obfuscation of the sample’s genome. Furthermore, if two samples are nearly identical in their genome sequence, then pooling their sequence data will do little to change the overall composition of the pool. Additional work is required to determine exactly how sequence diversity, genome size, and the number of pooled datasets affect how information is concealed with our method. To address this uncertainty, aligning reads to an incomplete reference genome (Figure 1A) can always be used to ensure that full reconstruction of a genome is not possible.

This work can be expanded to build standardized workflows for using incomplete reference genomes, as well as building variant call files and obfuscated consensus genomes from the pooled datasets. Any downstream applications using our data format will have equal or better data obfuscation than the pooled datasets. These data can also be used on the available platforms for pathogen data sharing (Griffiths et al., 2022; Karsch-Mizrachi et al., 2025; Shu and McCauley, 2017), however some adaptations may be required. For example, downstream obfuscated data would need to be clearly demarcated to ensure that users of the platform are aware that the sequence data had been obfuscated prior to analysis. Additionally, future iterations of this method could use the key file provided by our software to map specific reads back to their original datasets. This would permit full sample data retrieval when authorized, mirroring the functionality of molecular cryptography. Integrating our methods into these established data sharing platforms would offer a secure pathway to share high-risk pathogen data.

Many other types of genetic data are widely shared. For example, human genomic data can be shared to further research on human genomics or to develop new pharmaceutical drugs (Alvarellos et al., 2023). The sharing of human genomic data is complicated by issues of identifiability of the data (Gymrek et al., 2013) and the consequent violations of privacy (Bonomi et al., 2020; Erlich and Narayanan, 2014; McGuire, 2008). Human genetic data also has potential security issues that result from the fact that the people from whom the data is generated will be alive for decades and future technological advancements in biotechnology and AI may facilitate the misuse of this data (Sawaya et al., 2012). Like with pathogen data, concerns about the insecure sharing of human genomic data may ultimately lead to limitations on how data is shared.

The application of our data security techniques to other types of genomics data, like human data, represents a potential future direction of this work. Although pools made from larger genomes may require specialized variant calling pipelines to avoid false negatives, pooling genetic data has been theoretically proven to increase privacy regardless of genome complexity (Sawaya, 2017). Numerous techniques have been developed for human genetic data privacy and security (Oliva et al., 2024), including methods of genomic sequence obfuscation that utilize sequence similarity (Wan and Wang, 2022) or variant masking (Hekel et al., 2021). Other privacy and security methods can be applied to summary statistics obtained from human genomic data (Kamm et al., 2013; Wan et al., 2017). In contrast, our method is designed to obfuscate and mask dual-use genomic data for biosecurity applications. Furthermore, by operating on raw sequence data (FASTQ), our method can be integrated with downstream security techniques that operate on genome sequences or variants.

## Conclusion

The genomes analyzed here are of importance to biosecurity, and this method can have widespread applications to genomic data of other pathogens. This method can also be applied to larger genomes, such as human, to obfuscate sensitive data for privacy and security. Pooling genomic data, or equally, pooling genomic material, results in a mixture that prevents full reconstruction of a genome. This method, potentially combined with methods that remove some regions of the genome, can nevertheless allow useful genomic data to be shared for tasks such as population-level variant monitoring and pathogen surveillance (Park et al., 2025). The computational methods used here also have the potential to guide and optimize methods in molecular cryptography. The effectiveness of different pooling methods can be examined with this software, and the results can be used to design molecular cryptography.

By sharing raw data in a way that prevents misuse, analyses can be done to ensure an accurate interpretation of the data. The use of genetic data, in pathogen surveillance and elsewhere, can be essential for biosecurity, for the development of the bioeconomy, and the advancement of medicine. This data sharing can be stifled by the threat of misuse. The availability of raw data can help ensure the accuracy, authenticity and interpretation of any genomic analyses. Even when raw data is not to be shared, securing information within raw data itself provides security for the data at rest, and ensures that any information extracted from the data remains equally secure.

## Data Availability

The raw data supporting the conclusions of this article will be made available by the authors, without undue reservation.

## References

[B1] Alvarellos M. Sheppard H. E. Knarston I. Davison C. Raine N. Seeger T. (2023). Democratizing clinical-genomic data: How federated platforms can promote benefits sharing in genomics. *Front. Genet.* 13:1045450. 10.3389/fgene.2022.1045450 36704354 PMC9871385

[B2] Baker D. van den Beek M. Blankenberg D. Bouvier D. Chilton J. Coraor N. (2020). No more business as usual: Agile and effective responses to emerging pathogen threats require open data and open analytics. *PLoS Pathog* 16:e1008643. 10.1371/journal.ppat.1008643 32790776 PMC7425854

[B3] Bonomi L. Huang Y. Ohno-Machado L. (2020). Privacy challenges and research opportunities for genomic data sharing. *Nat. Genet.* 52 646–654. 10.1038/s41588-020-0651-0 32601475 PMC7761157

[B4] Buck M. Hamilton C. (2011). The nagoya protocol on access to genetic resources and the fair and equitable sharing of benefits arising from their utilization to the convention on biological diversity. *Rev. Eur. Commun. Intern. Environ. Law* 20 47–61. 10.1111/j.1467-9388.2011.00703.x

[B5] Chiara M. D’Erchia A. M. Gissi C. Manzari C. Parisi A. Resta N. (2020). Next generation sequencing of SARS-CoV-2 genomes: Challenges, applications and opportunities. *Brief Bioinform.* 22 616–630. 10.1093/bib/bbaa297 33279989 PMC7799330

[B6] Dokmanic I. Parhizkar R. Ranieri J. Vetterli M. (2015). Euclidean distance matrices: Essential theory, algorithms, and applications. *IEEE Signal Process. Magazine* 32 12–30. 10.1109/MSP.2015.2398954

[B7] Dos S Ribeiro C. Koopmans M. P. Haringhuizen G. B. (2018). Threats to timely sharing of pathogen sequence data. *Science* 362 404–406. 10.1126/science.aau5229 30361362

[B8] Doudna J. A. Charpentier E. (2014). Genome editing. The new frontier of genome engineering with CRISPR-Cas9. *Science* 346 1258096–1258096. 10.1126/science.1258096 25430774

[B9] Edelstein M. Sane J. (2015). *Overcoming Barriers to Data Sharing in Public Health: A Global Perspective.* London: Chatham House.

[B10] Edelstein M. Lee L. M. Herten-Crabb A. Heymann D. L. Harper D. R. (2018). Strengthening global public health surveillance through data and benefit sharing. *Emerg. Infect. Dis.* 24 1324–1330. 10.3201/eid2407.151830

[B11] Erlich Y. Narayanan A. (2014). Routes for breaching and protecting genetic privacy. *Nat. Rev. Genet.* 15 409–421. 10.1038/nrg3723 24805122 PMC4151119

[B12] Gibson D. G. Glass J. I. Lartigue C. Noskov V. N. Chuang R. Y. Algire M. A. (2010). Creation of a bacterial cell controlled by a chemically synthesized genome. *Science* 329 52–56. 10.1126/science.1190719 20488990

[B13] Griffiths E. J. Timme R. E. Mendes C. I. Page A. J. Alikhan N. F. Fornika D. (2022). Future-proofing and maximizing the utility of metadata: The PHA4GE SARS-CoV-2 contextual data specification package. *Gigascience* 11:giac003. 10.1093/gigascience/giac003 35169842 PMC8847733

[B14] Großwendt A. Röglin H. (2017). Improved analysis of complete-linkage clustering. *Algorithmica* 78 1131–1150. 10.1007/s00453-017-0284-6

[B15] Gymrek M. McGuire A. L. Golan D. Halperin E. Erlich Y. (2013). Identifying personal genomes by surname inference. *Science* 339 321–324. 10.1126/science.1229566 23329047

[B16] Hekel R. Budis J. Kucharik M. Radvanszky J. Pös Z. Szemes T. (2021). Privacy-preserving storage of sequenced genomic data. *BMC Genom.* 22:712. 10.1186/s12864-021-07996-2 34600465 PMC8487550

[B17] Huston P. Edge V. L. Bernier E. (2019). Reaping the benefits of open data in public health. *Can. Commun. Dis. Rep.* 45 252–256. 10.14745/ccdr.v45i10a01 31647060 PMC6781855

[B18] Juhas M. van der Meer J. R. Gaillard M. Harding R. M. Hood D. W. Crook D. W. (2009). Genomic islands: Tools of bacterial horizontal gene transfer and evolution. *FEMS Microbiol. Rev.* 33 376–393. 10.1111/j.1574-6976.2008.00136.x 19178566 PMC2704930

[B19] Kamm L. Bogdanov D. Laur S. Vilo J. (2013). A new way to protect privacy in large-scale genome-wide association studies. *Bioinformatics* 29 886–893. 10.1093/bioinformatics/btt066 23413435 PMC3605601

[B20] Karsch-Mizrachi I. Arita M. Burdett T. Cochrane G. Nakamura Y. Pruitt K. D. (2025). The international nucleotide sequence database collaboration (INSDC): Enhancing global participation. *Nucleic Acids Res.* 53 D62–D66. 10.1093/nar/gkae1058 39535044 PMC11701530

[B21] Karthikeyan S. Levy J. I. De Hoff P. Humphrey G. Birmingham A. Jepsen K. (2022). Wastewater sequencing reveals early cryptic SARS-CoV-2 variant transmission. *Nature* 609 101–108. 10.1038/s41586-022-05049-6 35798029 PMC9433318

[B22] Li P. E. Lo C. C. Anderson J. J. Davenport K. W. Bishop-Lilly K. A. Xu Y. (2017). Enabling the democratization of the genomics revolution with a fully integrated web-based bioinformatics platform. *Nucleic Acids Res.* 45 67–80. 10.1093/nar/gkw1027 27899609 PMC5224473

[B23] Lo C. C. Shakya M. Connor R. Davenport K. Flynn M. Gutiérrez A. M. Y. (2022). EDGE COVID-19: A web platform to generate submission-ready genomes from SARS-CoV-2 sequencing efforts. *Bioinformatics* 38 2700–2704. 10.1093/bioinformatics/btac176 35561186 PMC9113274

[B24] McGuire A. L. (2008). Identifiability of DNA data: The need for consistent federal policy. *Am. J. Bioeth.* 8 75–76. 10.1080/15265160802478511 19003718 PMC2771195

[B25] Mitchell L. A. Ellis T. (2017). Synthetic genome engineering gets infectious. *Proc. Natl. Acad. Sci. U. S. A.* 114 11006–11008. 10.1073/pnas.1715365114 29073011 PMC5651789

[B26] Murray-Rust P. (2008). Open data in science. *Nature Proc.* 3. 10.1038/npre.2008.1526.1 [Epub ahead of print].

[B27] Neves A. Cuesta I. Hjerde E. Klemetsen T. Salgado D. van Helden J. (2023). FAIR+E pathogen data for surveillance and research: Lessons from COVID-19. *Front. Public Health* 11:1289945. 10.3389/fpubh.2023.1289945 38074768 PMC10703184

[B28] Oliva A. Kaphle A. Reguant R. Sng L. M. F. Twine N. A. Malakar Y. (2024). Future-proofing genomic data and consent management: A comprehensive review of technology innovations. *Gigascience* 13:giae021. 10.1093/gigascience/giae021 38837943 PMC11152178

[B29] O’Toole Á Scher E. Underwood A. Jackson B. Hill V. McCrone J. T. (2021). Assignment of epidemiological lineages in an emerging pandemic using the pangolin tool. *Virus Evol.* 7:veab064. 10.1093/ve/veab064 34527285 PMC8344591

[B30] Park I. Kim Y. Choi M. H. Lee K. A. (2025). Genomic surveillance of SARS-CoV-2 variants using pooled WGS. *Sci. Rep.* 15:13948. 10.1038/s41598-025-99201-7 40263437 PMC12015266

[B31] Plotly (2024). *Dash Bio.Clustergram.* Montreal, QC: Plotly Technologies Inc.

[B32] Power J. J. Pinheiro F. Pompei S. Kovacova V. Yüksel M. Rathmann I. (2021). Adaptive evolution of hybrid bacteria by horizontal gene transfer. *Proc. Natl. Acad. Sci. U. S. A.* 118:e2007873118. 10.1073/pnas.2007873118 33649202 PMC7958396

[B33] Pratt B. Bull S. (2021). Equitable data sharing in epidemics and pandemics. *BMC Med. Ethics* 22:136. 10.1186/s12910-021-00701-8 34615519 PMC8493940

[B34] Rockett R. J. Arnott A. Lam C. Sadsad R. Timms V. Gray K. A. (2020). Revealing COVID-19 transmission in Australia by SARS-CoV-2 genome sequencing and agent-based modeling. *Nat. Med.* 26 1398–1404. 10.1038/s41591-020-1000-7 32647358

[B35] Rourke M. F. (2019). Access by design, benefits if convenient: A closer look at the pandemic influenza preparedness framework’s standard material transfer agreements. *Milbank Q.* 97 91–112. 10.1111/1468-0009.12364 30637812 PMC6422609

[B36] Rourke M. Eccleston-Turner M. (2021). The pandemic influenza preparedness framework as a “specialized international access and benefit-sharing instrument” under the Nagoya Protocol. *Northern Ireland Legal Quar.* 72 411–447. 10.53386/nilq.v72i3.881

[B37] Sawaya S. (2017). Cryptography for genetic material. *bioRxiv [Preprint]* 10.1101/157685

[B38] Sawaya S. Kenneally E. Nelson D. Schumacher G. (2012). *Artificial Intelligence and the Weaponization of Genetic Data.* Berlin: Springer eBooks.

[B39] Scarpa F. Sanna D. Azzena I. Cossu P. Locci C. Angeletti S. (2022). Genetic variability of the monkeypox virus clade IIb B.1. *J. Clin. Med.* 11:6388. 10.3390/jcm11216388 36362616 PMC9695420

[B40] Shu Y. McCauley J. (2017). GISAID: Global initiative on sharing all influenza data - from vision to reality. *Euro Surveill.* 22:30494. 10.2807/1560-7917.ES.2017.22.13.30494 28382917 PMC5388101

[B41] Smith J. A. Sandbrink J. B. (2022). Biosecurity in an age of open science. *PLoS Biol.* 20:e3001600. 10.1371/journal.pbio.3001600 35421093 PMC9009689

[B42] Smith J. T. Andam C. P. (2021). Extensive horizontal gene transfer within and between species of coagulase-negative *Staphylococcus*. *Genome Biol. Evol.* 13:evab206. 10.1093/gbe/evab206 34498042 PMC8462280

[B43] Thompson A. K. Faith K. Gibson J. L. Upshur R. E. (2006). Pandemic influenza preparedness: An ethical framework to guide decision-making. *BMC Med. Ethics* 7:E12. 10.1186/1472-6939-7-12 17144926 PMC1698926

[B44] Vinatzer B. A. Heath L. S. Almohri H. M. J. Stulberg M. J. Lowe C. Li S. (2019). Cyberbiosecurity challenges of pathogen genome databases. *Front. Bioeng. Biotechnol.* 7:106. 10.3389/fbioe.2019.00106 31157218 PMC6529814

[B45] Wan S. Wang J. (2022). A sequence obfuscation method for protecting personal genomic privacy. *Front. Genet.* 13:876686. 10.3389/fgene.2022.876686 35495121 PMC9043694

[B46] Wan Z. Vorobeychik Y. Kantarcioglu M. Malin B. (2017). Controlling the signal: Practical privacy protection of genomic data sharing through Beacon services. *BMC Med. Genom.* 10 (Suppl. 2):39. 10.1186/s12920-017-0282-1 28786360 PMC5547445

