# Supplementary material for: Methods for safely sharing dual-use genetic data
Source: Front Microbiol. 2026 Feb 11;17:1716431. doi: 10.3389/fmicb.2026.1716431 (PMC12932512; doi:10.3389/fmicb.2026.1716431)

Supplementary Figure 1. *B.anthracis* Pooled 5-samples SNVs and individual sample SNVs

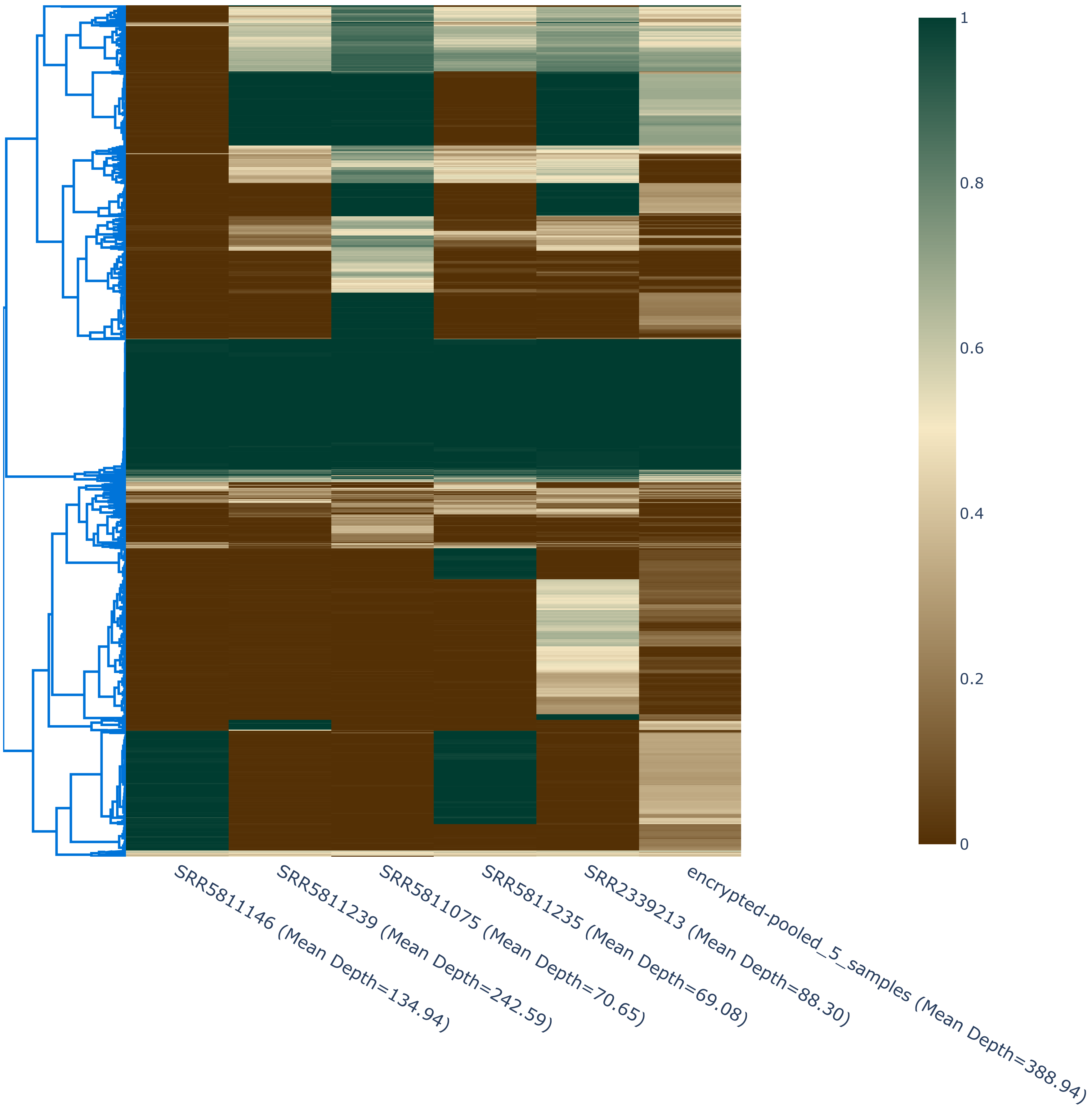

Supplementary Figure 2. *B.anthracis* Pooled 10-samples SNVs and individual sample SNVs

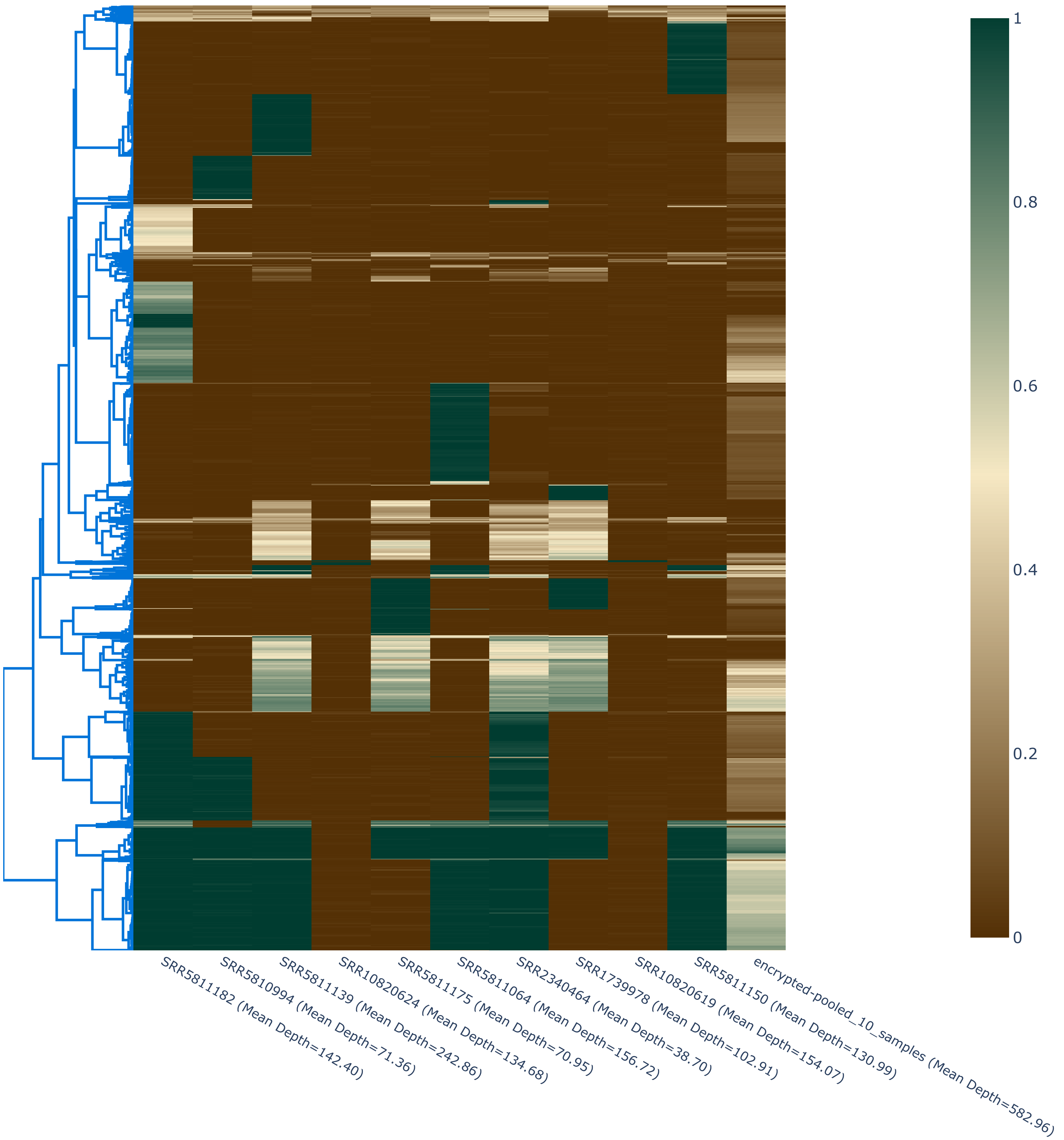

Supplementary Figure 3. *B.anthracis* Pooled 50-samples SNVs and individual sample SNVs

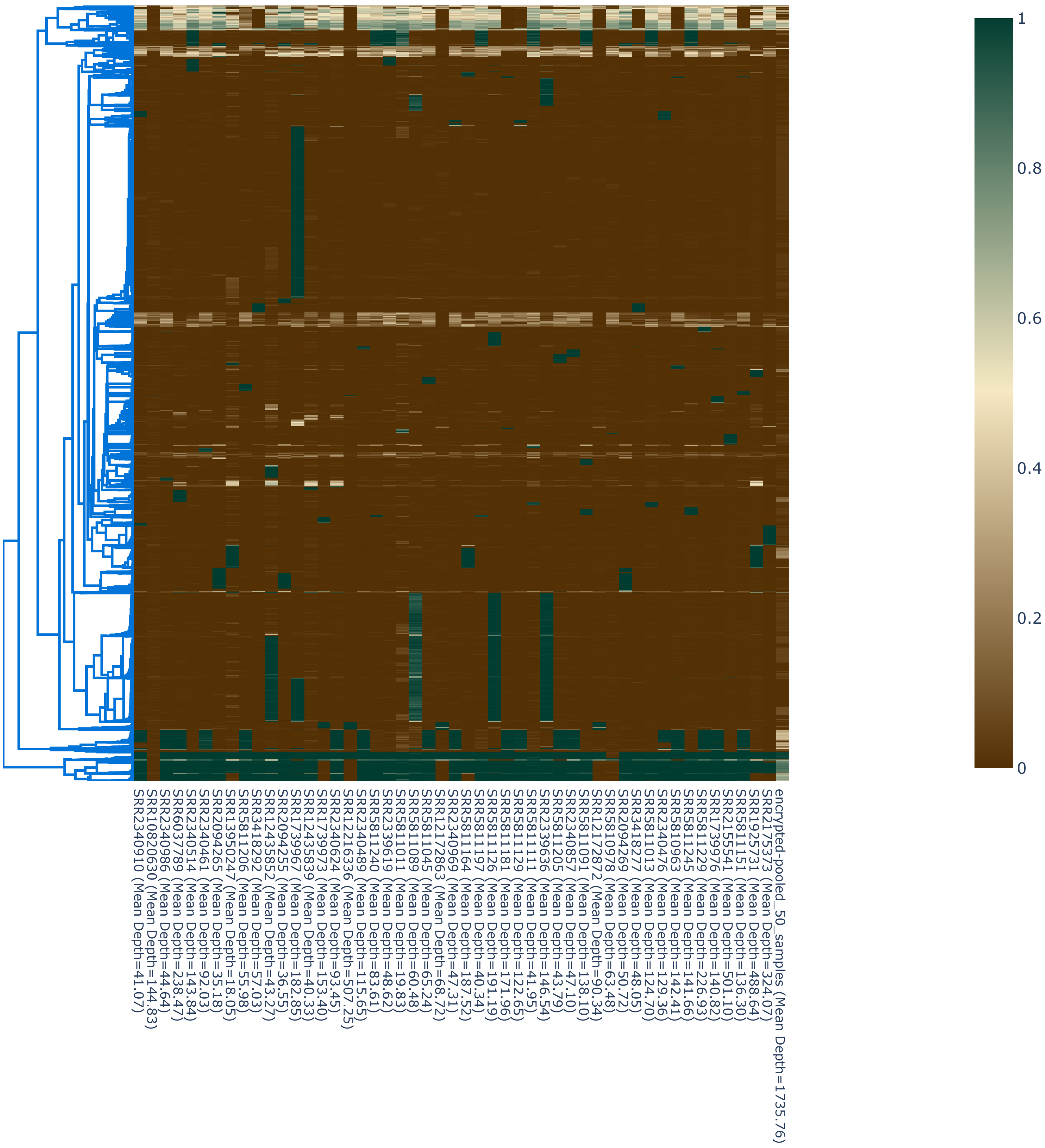

Supplementary Figure 4. Monkeypox virus pooled 5-samples SNVs and individual sample SNVs

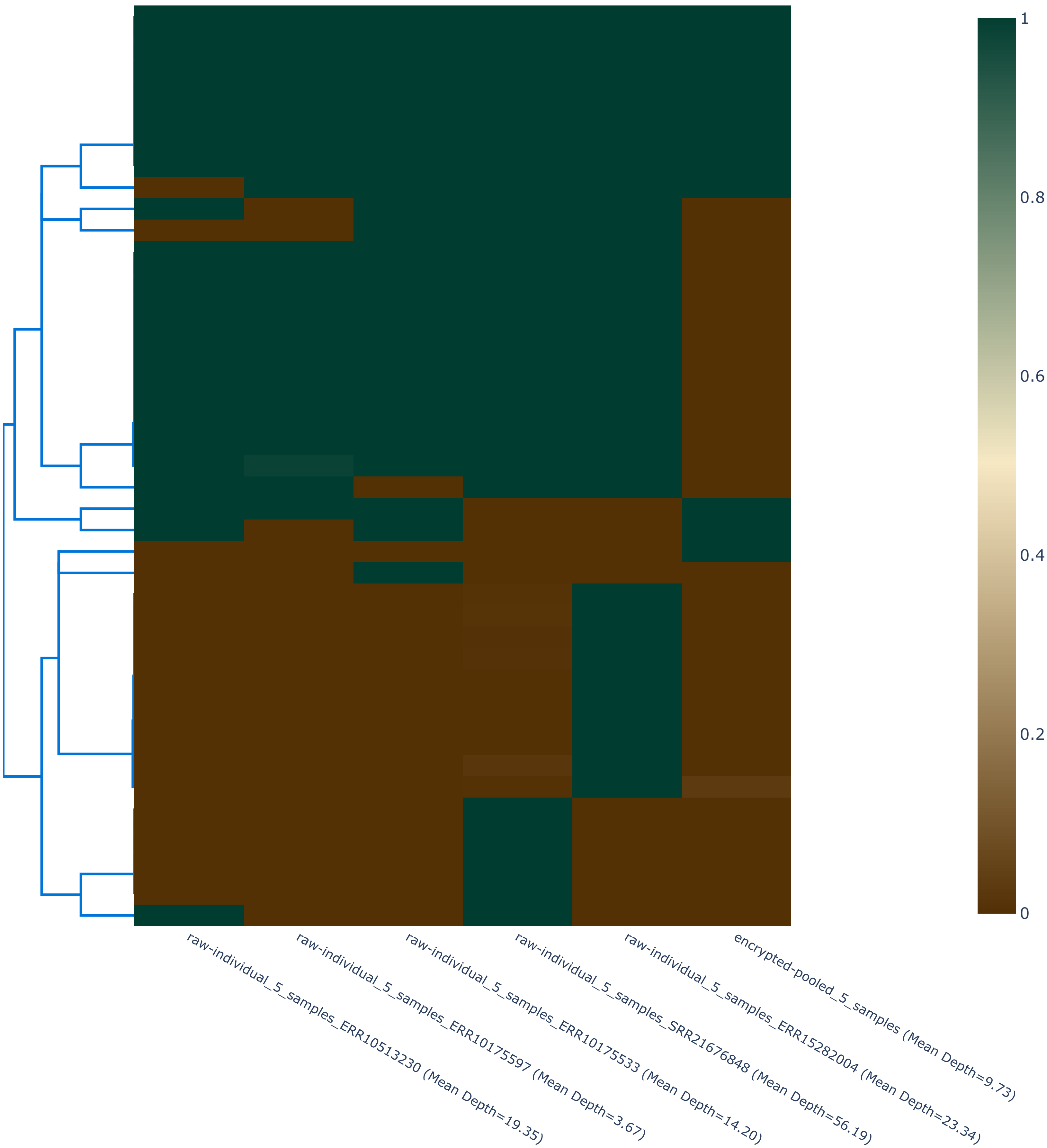

Supplementary Figure 5. Monkeypox virus pooled 10-samples SNVs and individual sample SNVs

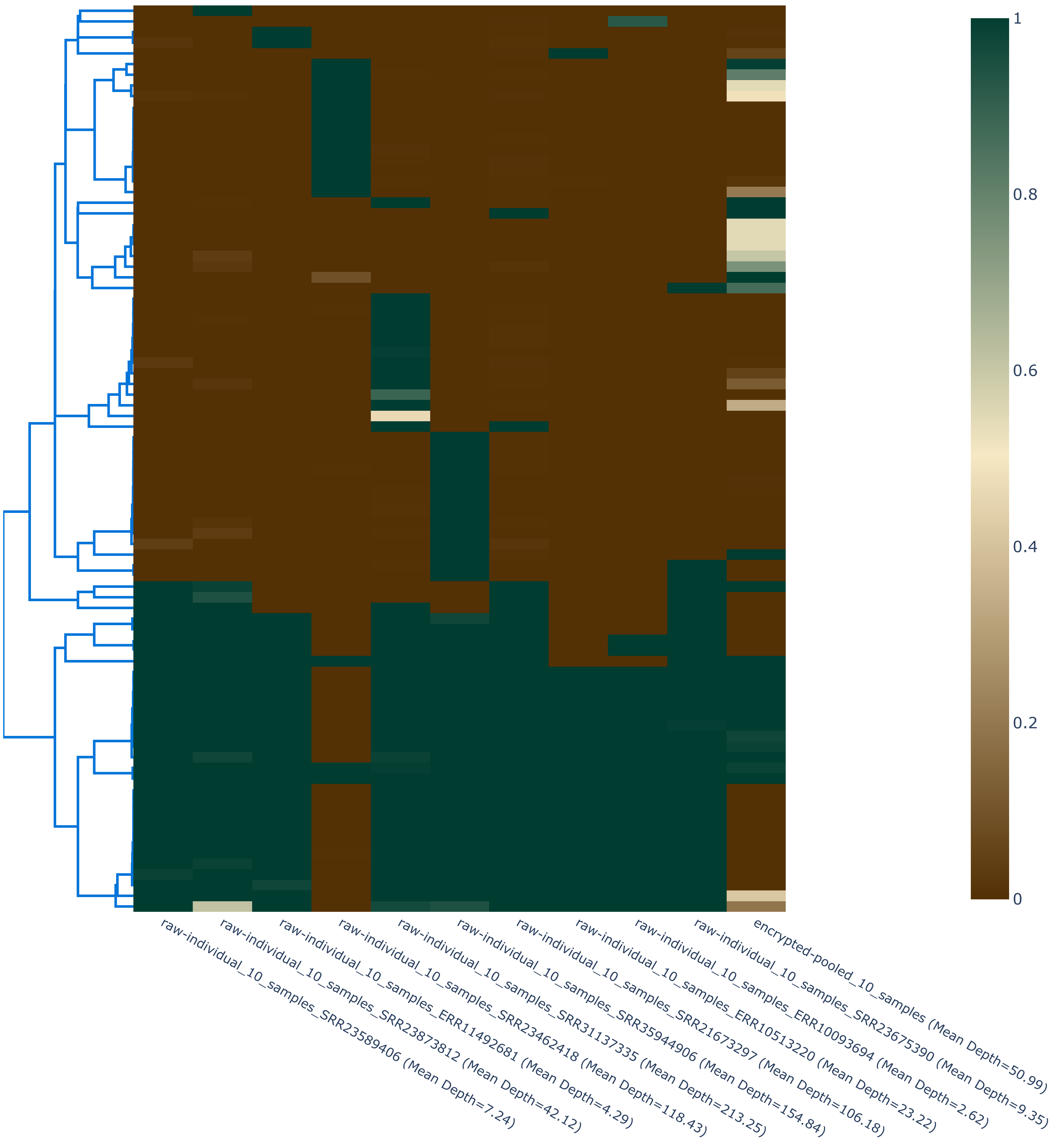

Supplementary Figure 6. Monkeypox virus pooled 50-samples SNVs and individual sample SNVs

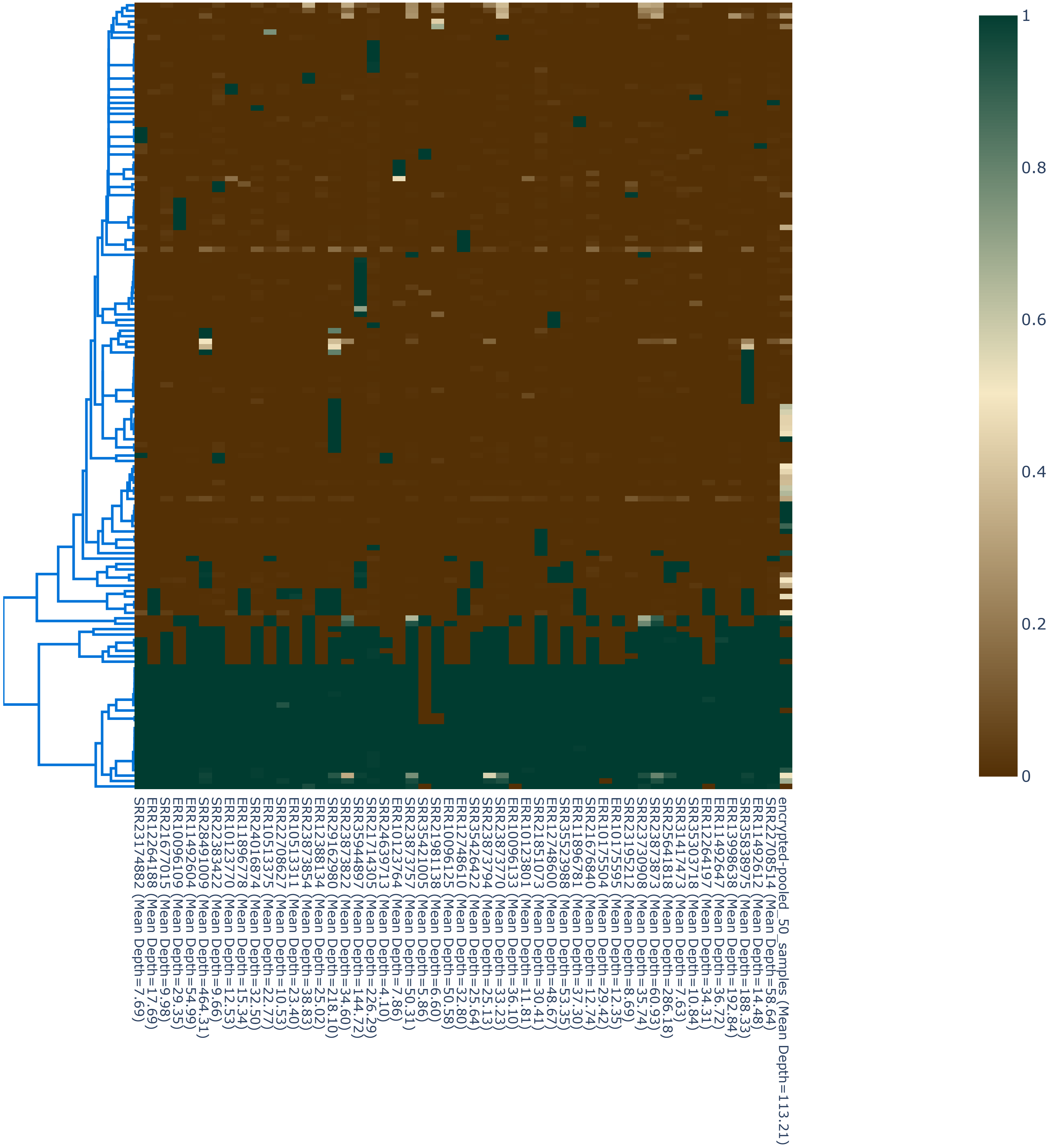

Supplement: Supplementary file 1 [file Data_Sheet_1.pdf]
